# The effects of moderate alcohol supplementation on estrone sulfate and DHEAS in postmenopausal women in a controlled feeding study

**DOI:** 10.1186/1475-2891-3-11

**Published:** 2004-09-07

**Authors:** Somdat Mahabir, David J Baer, Laura L Johnson, Joanne F Dorgan, William Campbell, Ellen Brown, Terryl J Hartman, Beverly Clevidence, Demetrius Albanes, Joseph T Judd, Philip R Taylor

**Affiliations:** 1 Cancer Prevention Studies Branch, Center for Cancer Research, National Cancer Institute, Bethesda, MD, USA; 2 Population Science Division, Fox Chase Cancer Center, Philadelphia, PA, USA; 3 Human Nutrition Research Center, Agriculture Research Service, U.S. Department of Agriculture, Beltsville, MD, USA; 4 Department of Nutrition, Pennsylvania State University, University Park, PA, USA; 5 Nutritional Epidemiology Branch, Division of Cancer Epidemiology and Genetics, National Cancer Institute, Bethesda, MD, USA

**Keywords:** Alcohol, Hormones, Postmenopausal women

## Abstract

**Background:**

We have demonstrated that moderate alcohol consumption (15 g/d, 30 g/d) for 8 weeks resulted in significantly increased levels of serum estrone sulfate and DHEAS in 51 postmenopausal women in a randomized, placebo-controlled trial. We now report on the relationships between serum estrone sulfate and dehydroepiandrosterone sulfate (DHEAS) levels after 4 weeks of moderate alcohol supplementation, and compare the results to the 8 weeks data to elucidate time-to-effect differences.

**Methods:**

Postmenopausal women (n = 51) consumed 0 (placebo), 15 (1 drink), and 30 (2 drinks) g alcohol (ethanol)/ day for 8 weeks as part of a controlled diet in a randomized crossover design. Blood samples were drawn at baseline, at 4 weeks and at 8 weeks. Changes in estrone sulfate and DHEAS levels from placebo to 15 g and 30 g alcohol per day were estimated using linear mixed models.

**Results and Discussion:**

At week 4, compared to the placebo, estrone sulfate increased an average 6.9% (P = 0.24) when the women consumed 15 g of alcohol per day, and 22.2% (P = 0.0006) when they consumed 30 g alcohol per day. DHEAS concentrations also increased significantly by an average of 8.0% (P < 0.0001) on 15 g of alcohol per day and 9.2% (P < 0.0001) when 30 g alcohol was consumed per day. Trend tests across doses for both estrone sulfate (P = 0.0006) and DHEAS (P < 0.0001) were significant. We found no significant differences between the absolute levels of serum estrone sulfate at week 4 versus week 8 (P = 0.32) across all doses. However, absolute DHEAS levels increased from week 4 to week 8 (P < 0.0001) at all three dose levels.

**Conclusions:**

These data indicate that the hormonal effects due to moderate alcohol consumption are seen early, within 4 weeks of initiation of ingestion.

## Background

Epidemiological evidence consistently shows a positive association between alcohol, even low to moderate intake, and breast cancer risk [1]. However, during the past two decades, it has become evident that moderate drinking is associated with longer life [2], reduced rates of heart disease [3] and stroke [4]. What does this mean for women when the epidemiologic data show an exposure is associated with both benefits and harms? Recommendations regarding the use or avoidance of moderate alcohol, must take into consideration both its potential benefit on cardiovascular disease, as well as its potential risk for breast cancer. To understand the biologic parameters potentially influenced by alcohol, there is a need for well-controlled mechanistic studies of dose and duration of use on markers of risk in the causal pathway of breast cancer.

A reanalysis of nine prospective studies shows that levels of endogenous sex hormones are strongly associated with breast cancer risk in postmenopausal women [5]. Clear evidence that moderate alcohol consumption increases levels of hormones associated with increased risk for breast cancer would provide support for a causal relationship. In a controlled study of acute alcohol ingestion, serum estrone levels were significantly elevated in postmenopausal women on hormone replacement therapy (HRT), but did not affect serum estrone levels in women not using HRT [6]. To better understand the effects of moderate long-term alcohol ingestion on sex hormones, we conducted a controlled feeding study in postmenopausal women not using HRT. As previously reported, there were significant elevations in both serum estrogen sulfate and dehydroepiandrosterone sulfate (DHEAS) after 8 weeks of supplementation [7]. Here, we evaluate the relationships between serum estrogen sulfate and DHEAS after 4 weeks of supplementation with moderate alcohol, and compare the results to the 8 week data to elucidate time-to-effect differences.

## Methods

Details of the Women's Alcohol Study (WAS) was previously published [7]. Briefly, a total of 51 postmenopausal women not on HRT completed the study and are included in the analysis. The WAS utilized a crossover design. Each participant rotated through three 8-weeks controlled dietary periods during which she consumed a beverage daily that contained no alcohol (placebo), 15 g alcohol, or 30 g alcohol in random order. Each of the three dietary periods was preceded by a 2- to 5-week washout period during which the women consumed no alcohol. Alcohol was supplied as 95% ethanol (Everclear™ Pharmaco Products, Inc., Brookfield, CN) in orange juice (12 ounces). All meals were prepared at the Beltsville Human Nutrition Research Center and the participants ate breakfast and supper at the Center and had carryout lunches on weekdays. On weekends, food and beverages were packaged for consumption at home. The calorie level for each subject was adjusted to maintain constant body weight.

Blood for hormone analyses was collected after an overnight fast at weeks 4 and 8. Serum was separated, and aliquots were frozen at -70°C. In the samples taken after 4 weeks of alcohol supplementation, we only measured serum estrone sulfate and DHEAS, as they were the only two hormones that were significantly elevated at 8 weeks [7].

Hormone concentrations were transformed using natural log. Changes in hormone concentrations from placebo to 15 g and 30 g of alcohol per day were estimated at 4 and 8 weeks using linear mixed models including a random intercept and alcohol levels as fixed effects treated as two indicator variables. Separate models used alcohol as a continuous variable to test for trend. Regressions to evaluate the effect of several baseline covariates on the precision of the parameter estimates for alcohol consumption included age, BMI, and years since menopause modeled as continuous fixed effects; assignment order, dietary period, hysterectomy, and race were modeled as indicator variables. Effect modification by race, assignment order, dietary period, age, BMI, and years since menopause were assessed by likelihood ratio tests of improvement in the model fit after addition of cross-product terms to models that included main effects for alcohol and the characteristic being evaluated. All tests of statistical significance were two-sided. Statistical analyses were performed using S-PLUS (S-PLUS version 6.1 for Windows. Seattle (WA): Insightful Corporation; 2002.)

## Results and Discussions

Table 1 summarizes the mean hormone concentrations at four weeks and eight weeks in the participants when not consuming alcohol and the percent changes from no alcohol consumption when consuming 15 g or 30 g of alcohol per day, respectively. At 4 weeks of alcohol intake the inclusion of age, years since menopause, race, and baseline BMI in the models did not change the precision of parameter estimates for alcohol doses and thus results from simple models are presented. At week 4, compared to the no alcohol placebo, estrone sulfate increased an average 6.9% (P = 0.24) when women consumed 15 g of alcohol per day and 22.2% (P = 0.0006) when they consumed 30 g of alcohol per day. DHEAS concentrations also increased significantly by an average 8.0% (P < 0.0001) on 15 g of alcohol per day and 9.2% (P < 0.0001) when 30 g alcohol was consumed per day at week 4. Trend tests for both estrone sulfate (P = 0.0006) and DHEAS (P < 0.0001) were highly significant.

**Table 1:** Geometric mean hormone levels (ng/dL) for participants on 0 g alcohol and % change (Δ) in hormone levels from 0 g to 15 g and 30 g alcohol per day at weeks 4 and 8

Hormone		0 g/d, mean (95% C.I.)	15 g/d, Δ (95% C.I.)*	30 g/d, Δ (95% C.I.)*	*P*-trend†
Estrone Sulfate	(4 wk)	47.5 (39.2–57.4)	6.9% (-4.2%–19.3%)	22.2% (9.4%–36.5%)	0.0006
	(8 wk)	47.4 (40.5–55.6)	7.5% (-0.3%–15.9%)	10.7% (2.7%–19.3%)	0.009
DHEAS	(4 wk)	55.3 (47.0–65.1)	8.0% (4.5%–11.6%)	9.2% (5.6%–12.8%)	<0.0001
	(8 wk)	59.4 (50.5–70.0)	5.1% (1.4%–9.0%)	7.5% (3.7%–11.5%)	0.0001

* Estimates of percent change are from linear mixed models, including participant as a random effect and alcohol levels as fixed effects treated as two indicator variables.

† *P*-trend values (two-sided) are from linear mixed models, including participant as a random effect and alcohol levels as a continuous fixed effect with values 0, 15, and 30.

The effects of alcohol supplementation on serum estrone sulfate and DHEAS levels did not vary with age, BMI, race, or years since menopause. These hormone concentrations also did not differ among the three dietary periods, and the order of the assignment to the treatment regimens did not modify the associations with either of the two hormones.

At 8 weeks of alcohol intake, the 15 g dose versus the placebo increased serum estrone sulfate and DHEAS by 7.5% and 5.1% respectively, whereas the 30 g dose compared to the placebo, increased levels of estrone sulfate and DHEAS by 10.7% and 7.5% respectively. When comparisons using linear mixed models are made, after controlling for alcohol intake across all doses, there was no statistically significant difference between the absolute levels of serum estrone sulfate at week 4 versus 8 (P = 0.32). However, controlling for alcohol intake absolute DHEAS levels increased between weeks 4 and 8 (P < 0.0001). The increase in DHEAS between weeks 4 and 8 did not occur in just the 15 or 30 g per day groups, however; the increase was similar in each of the three groups. In the 0 g per day group the geometric mean shifted from 55.3 ng/dL at 4 weeks to 59.4 ng/dL at 8 weeks. In the 15 g per day group the geometric mean shifted from 59.7 ng/dL to 62.5 ng/dL, and in the 30 g per day group the geometric mean shifted from 60.4 ng/dL to 63.9 ng/dL. The models did not suggest an interaction between measurement week and alcohol dose in serum estrone sulfate (P = 0.32) or DHEAS (P = 0.58).

In postmenopausal women moderate alcohol consumption for four weeks resulted in statistically significant increased levels of serum estrone sulfate, the most abundant circulating estrogen, and DHEAS, the steroid hormone with the highest concentration in the blood. Compared to the placebo, serum estrone sulfate levels increased 6.9% (P = 0.24) and 22.2% (P = 0.0006) respectively among women who consumed 15 g and 30 g alcohol per day for four weeks. Serum DHEAS concentrations also increased by 8.0% (P < 0.0001) and 9.2% (P < 0.0001) respectively among women who consumed 15 g or 30 g alcohol per day for four weeks. At week 8, serum estrone sulfate levels increased 7.5% and 10.7% respectively among women who consumed 15 g and 30 g alcohol per day whereas DHEAS concentrations increased 5.1% and 7.5% respectively among women who consumed 15 g or 30 g alcohol per day compared to the placebo [7]. The increased levels of serum estrone sulfate concentrations at week 4 were essentially the same as those seen at week 8. The % change in serum estrone sulfate levels from week 4 to week 8 is -0.1%, 0.5%, and -8.9% respectively in the 0 g, 15 g, and 30 g alcohol doses. Estrone sulfate does not show a consistent change over time across all alcohol doses and there is no statistical evidence of an interaction between time and alcohol (P = 0.32). Although there appears to be a difference when looking at the point estimates in the 30 g alcohol dose, variance in estrone sulfate is so large at both 4 weeks and 8 weeks the difference between the two point estimates is not statistically significant. The large variance in estrogen sulfate at the 30 g dose will require more people to accurately judge this possible interaction.

For DHEAS, the absolute levels were statistically (P < 0.0001) higher at week 8 compared to week 4 in all the alcohol groups. This increase in DHEAS between weeks 4 and 8 did not differ regardless of 0 g, 15 g, or 30 g per day dose (P = 0.58). Biological reasons may explain the increase in the 15 g and 30 g per day alcohol dose, but the reasons behind the increases in DHEAS for the 0 g dose need further research. We did not find any evidence for an effect modification between measurement week and alcohol supplementation in serum estrone sulfate (P = 0.32) or DHEAS (P = 0.58).

These data indicate that the hormonal effects due to moderate consumption of alcohol equivalent to one or two drinks per day are seen early, within 4 weeks of initiation of ingestion. Importantly from a study design perspective, our study also demonstrates that it may be possible to utilize shorter study periods when assessing the effects of alcohol consumption on hormone levels, at least in postmenopausal women. To understand the earliest effects of moderate alcohol intake on hormone levels in postmenopausal women, future studies will have to be designed to assess serum levels earlier than 4 weeks or possibly later than 8 weeks.

Postmenopausal women with elevated levels of serum estrone sulfate [8,9] and DHEAS [8-11] levels were reported to be at an increased risk of breast cancer in several prospective cohort studies. Results from our study showing statistically significant increased serum estrone sulfate and DHEAS concentrations after four weeks of supplementation with alcohol equivalent to one or two drinks per day, provide one possible mechanism by which moderate alcohol ingestion could increase breast cancer risk in postmenopausal women.

Alcohol has many physiologic effects and could influence breast cancer risk through non-hormonal mechanisms as well. Experimental evidence suggests that alcohol interferes with folate absorption, transport, and metabolism, potentially limiting folate stores in the tissues and may interfere with DNA methylation [12,13]. Alcohol consumption and metabolism can result in increased production of several classes of DNA damaging molecules including reactive oxygen species [14] which can lead to increase DNA damage and the development of breast cancer [15].

## Conclusions

In conclusion, our results provide additional evidence for a mechanism by which moderate alcohol drinking could modify breast cancer risk, indicating that this effect occurs after a short time period (as early as four weeks), and thus provides further support for a causal association.

## Competing interests

None declared.
